# Supplementary material for: The effect of journal guidelines on the reporting of antibody validation
Source: PeerJ. 2020 Jun 3;8:e9300. doi: 10.7717/peerj.9300 (PMC7275675; doi:10.7717/peerj.9300)
Supplement: Article S1 [file peerj-08-9300-s001.docx]

# Journal guidelines - *Journal of Comparative Neurology (JCN)*

The leading journal when it comes to reporting on antibodies in scientific articles is the *Journal of Comparative Neurology.* This journal was the first one to generate attention for the problems related to antibodies and antibody reporting and it is responsible for what some people call the ‘one-journal revolution’ on antibody reporting (Baker 2015).

On 25 August 2003, *JCN* published an editorial explaining the minimal types of validation that should be carried out with antibodies used in immunohistochemistry. In this editorial, *JCN* also informed its authors that scientific articles with antibodies in which the antigen is not reported would no longer be published. It also encourages authors to provide full information on the antibodies used; authors need to provide both the catalog number and information on which batch was used. This editorial mostly created awareness of the problems that can arise when using antibodies. It did not provide strict guidelines on antibody reporting in *JCN (Saper & Sawchenko 2003).*

Two years later, on 22 November 2005, Clifford Saper, the editor in chief of *JCN* from 1994 to 2011, published another editorial commenting on the use of antibodies in research. The editors of *JCN* had become aware of retractions caused by problems with antibodies. This editorial describes a set of rules that should from then onwards be applied to every paper using antibodies (Saper 2005).

After that editorial, *JCN* implemented antibody reporting guidelines, which can be found under the instructions for authors section on the journal’s website. With regard to antibodies, they require authors to add an ‘Antibody Characterization’ section to the methods section of a paper. They also state that they support the Resource Identification Initiative (RRID). Information retrieved from the Author Guidelines section of website *Journal Comparative Neurology* on 04-05-2018.


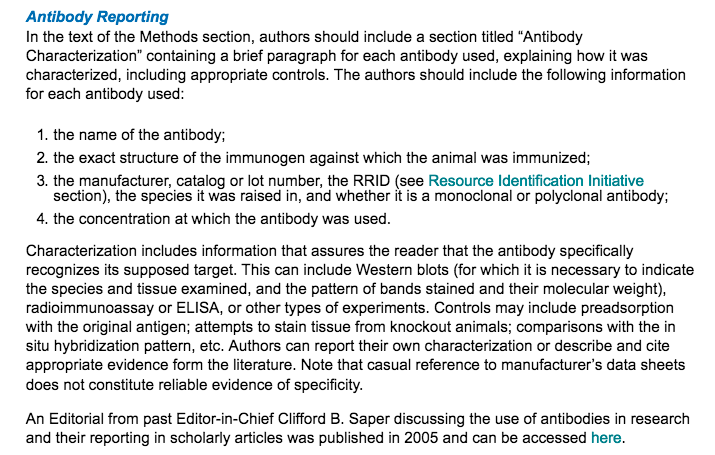


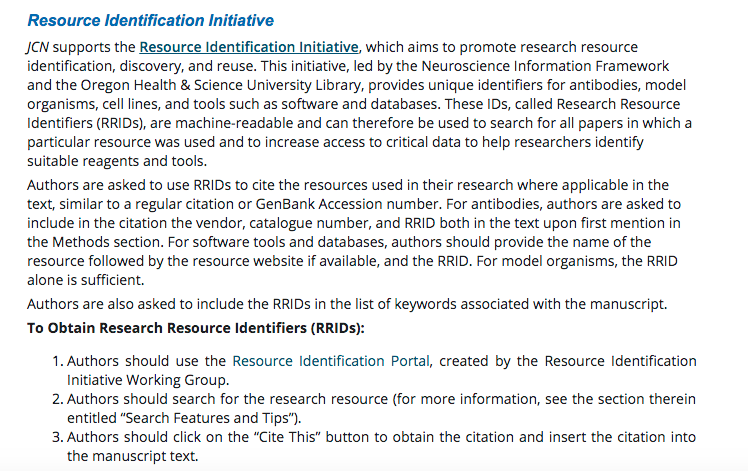


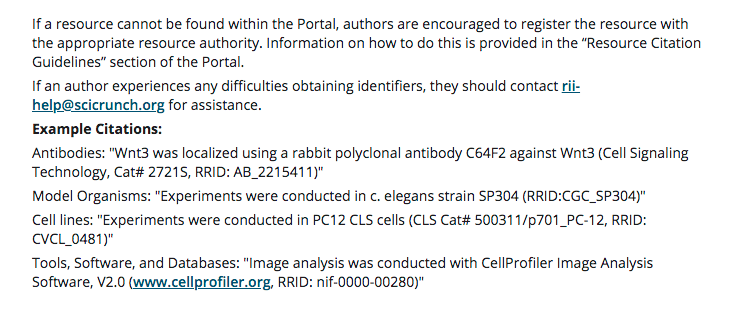


When authors want to submit a manuscript to *JCN,* they need to check a box that states that their publication provides full information on all antibodies used. Submission process tested on 09-05-2018).


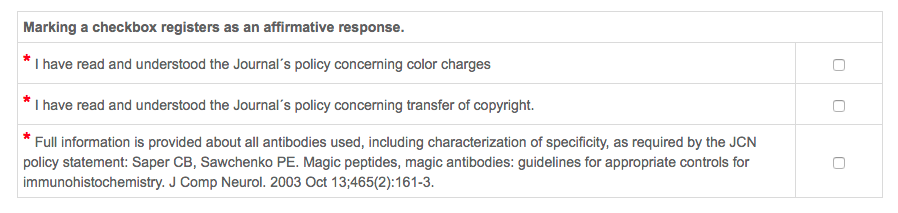


# Journal guidelines - *Nature*

In an effort to reduce the irreproducibility of the research published in their journal, *Nature* implemented a Reporting Checklist for Life Science Articles in May 2013. This checklist should improve reporting of a few crucial experimental and analytical design elements of research. From that moment onwards, authors were required to fill out this checklist at submission of their paper. At the same time, *Nature* also abolished space restrictions on the methods section (Nature 2013)*.*

Antibody section of Reporting Checklist for Life Sciences Articles 2013:

*
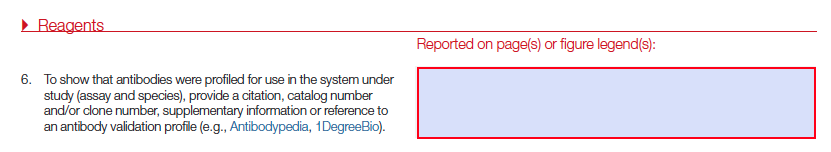
*

On 1 June 2017, *Nature* published another announcement stating that the journal would go one step further in trying to improve the quality of reporting. From then onwards, authors in the life science field are required to fill out a Life Science Reporting Summary that will be published as supplementary information alongside the article. This document contains details about the experimental design, analysis and reagents (Nature 2017).

Antibody section of Reporting Summary for Life Sciences Articles June 2017:

*
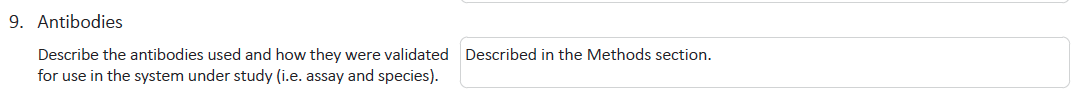
*

Antibody section of Reporting Summary for Life Sciences Articles March 2018


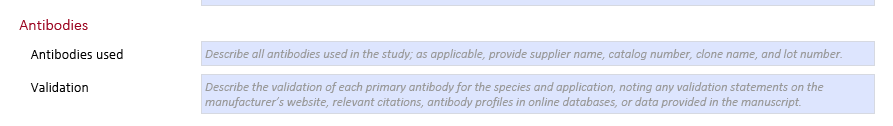


During the submission process, *Nature* asks its authors to upload the reporting summary at the same moment as the manuscript. The journal warns its authors that failure to hand it in at submission may delay the handling of the submission since the form needs to be provided to reviewers if the paper is sent for peer-review (submission process tested on 09-05-2018).

# References

Baker M. 2015. Antibody anarchy: A call to order. *Nature* 527:545. 10.1038/527545a

Nature. 2013. Reducing our irreproducibility. *Nature* 496:398-398.

Nature. 2017. Towards greater reproducibility. *Nature* 546. 10.1038/546008a

Saper CB. 2005. An open letter to our readers on the use of antibodies. *The Journal of Comparative Neurology* 493:477-478. 10.1002/cne.20839

Saper CB, and Sawchenko PE. 2003. Magic peptides, magic antibodies: guidelines for appropriate controls for immunohistochemistry. *Journal of Comparative Neurology* 465:161-163.
