# Supplementary material for: The effect of journal guidelines on the reporting of antibody validation
Source: PeerJ. 2020 Jun 3;8:e9300. doi: 10.7717/peerj.9300 (PMC7275675; doi:10.7717/peerj.9300)
Supplement: Article S2 — Protocol used to extract the antibody validation information from the evaluated articles. [file peerj-08-9300-s002.docx]

# 1 - Coding protocol

Validation information was collected from the sampled articles and all supplementary information (SI) provided with these articles and then noted down in a table (see 2 - Antibody validation coding table). Vendor websites, antibody databases, or other secondary scientific literature were not used to further determine how well an antibody was validated. The scientific articles were used as the unit of analysis, not the antibodies. This means that the reporting of antibody validation in the articles was coded per article and not separately for each antibody that was used in an article. Validation information was marked as being present if the validity of at least one antibody in a paper could be established.

A paper can describe the use of different methods of validation for the different antibodies that were used. In that case all different methods of validation used in the paper were noted. For example, a paper can cite the literature as proof of validation for one antibody and provide positive and negative controls for another antibody used. In this case, both methods of validation were noted. Furthermore, it was also noted if different methods of validation were used for different antibodies, if validation information was not reported for all antibodies, if multiple types of validation were used for one antibody, and if all primary antibodies had been validated. This was done to provide a more elaborate picture of how extensively validation was carried out.

To extract the validation information, the articles as well as all SI and extra figures were scanned using the word ‘anti‘ to find the sections where the use of antibodies is described. The papers were also scanned for use of the word ‘validation’ in relation to antibodies or words related to validation, such as ‘characterization’, ‘validate’, ‘specificity’ and ‘reliability’. Furthermore, all figures were checked for the use of antibodies, because antibodies are sometimes used without being described in the caption of a figure. The antibody information found in these areas of the paper was then used to fill out the coding table. If the description of antibodies or the experiments was unclear, additional parts of the paper were consulted, and in some cases the entire paper was read.

Antibody validation was assessed by answering ‘yes’ or ‘no’ questions in a coding table. In case it was unclear a ‘?’ was filled out, and in case the question was not applicable (for example the questions about type of validation when no antibodies were validated) ‘na’ was filled out. When evaluating the articles, first, the question was answered whether the validity of at least one of the antibodies used could be established by reading the paper or SI. Antibody validation can purposefully be carried out, meaning that experiments were carried out with the purpose of establishing the validity of an antibody. However, when evaluating the articles often the validity of an antibody could be established from the experiments it was used in (for example when a wild type and knock out sample were both stained with the same antibody). No distinction was made between purposeful validation and possible ‘accidental’ validation, as we cannot know the intentions of the authors. In both cases the antibody (and thus the scientific article it was used in) was marked as being validated.

After this, the types of validation used in an article were assessed. First, a distinction was made between validation carried by researchers themselves and validation carried out by third parties. In this last category, a distinction was made between validation by reference to the literature, to information of the supplier, or to information in online databases. In these cases, the authors of an article explained that the antibody had previously been validated by this third party and/or provided a reference to where the antibody validation information could be found. The type of validation carried out by this third party was not further specified, but it can consist of any of the types of validation described in Appendix 3 - Methods of antibody validation.

In contrast, the category ‘validation by researchers themselves’ was then further specified into different methods of validation. These different categories of antibody validation were chosen after studying the literature on antibody validation, by performing coding trials to extract validation information from published articles, and by having conversations with scientists working frequently with antibodies. There might be some overlap in different validation methods. This is the case with the five pillar methods for example. When the method genetic strategies is used, this often means that a positive and negative control were used. Another example could be orthogonal strategies, which could mean that RNA based methods were used. In these cases, all methods are written down. Section 3 - Methods of antibody validation provides a comprehensive list of types of validation. Section 4 - Coding examples shows an example of how coding decisions were made with regard to antibody validation.

Apart from antibody validation, antibody information was also assessed. The basic antibody information for commercial antibodies was ranked as ‘complete’ if either an RRID or the name, supplier and catalog number of all antibodies used in a study were reported. For non-commercial antibodies either an RRID or the host-animal and immunogen used needed to be reported. If a paper only provides these characteristics of some antibodies and not of others, the antibody information of the paper was not considered to be complete.

In case there was doubt while filling in the coding table, decisions were always made that would be most favorable to the article. Examples could be doubt about if an antibody was validated or not or doubt about how many antibodies were validated. In the latter case, the highest number would be written down to give the article the benefit of the doubt. Any doubts or unusual observations were explained in the ‘comments’ section of the table.

# 2 - Antibody validation coding table

Questions were filled out with yes (1), no (0), not clear (999), or not applicable (9)

| Part 1. Questions answered for all evaluated articles | | |
| --- | --- | --- |
| Category | **Question about article** | **Further explanation** |
| Information article | DOI: |  |
|  | URL: |  |
|  | Journal: |  |
|  | Times cited: | At date retrieval. |
|  | Date published: | Date from Web of Science. |
|  | Date received: | Date from website article. |
|  |  |  |
| Antibody information | Is a section devoted to antibodies present in the article? | If the word antibody is mentioned above a paragraph about antibodies, this question is answered with yes. E.g. the antibody section in Life Science Reporting Summary. |
|  | How many different antibodies are used? | Look at article and all its supporting information, extra figures, tables etc. |
|  | Which applications using antibodies are used? |  |
|  | Are commercial antibodies used? |  |
|  | Are antibodies used that are generated by the researchers themselves or obtained from other researchers? |  |
|  | Is any antibody information (characteristics) present? | Any antibody information. E.g. if only catalog no. is given this question is answered with yes. |
|  | Is the basic antibody information of all commercial antibodies complete? | Complete means at least: name, supplier, and catalog no. Alternatively an RRID might be provided. |
|  | Is the basic antibody information of all non-commercial antibodies complete? | Complete means at least: host-animal and immunogen. Alternatively, an RRID might be provided. |
|  | Is the basic information of all primary antibodies complete? | Both commercial and non-commercial antibodies. |
|  |  |  |
| Validation | Is any validation information about the antibodies present? | Any validation info present for at least one antibody. For example:   - A statement such as ‘the specificity of antibody X was verified by pre-adsorption,’ means this question is answered with yes. - An experiment in which a positive or negative control is shown that would validate the antibody even if it was never the purpose to validate it means this question is answered with yes. - An experiment in which an antibody is used to stain a target protein in cell lines in which this target protein is brought to expression but would not naturally occur means the question is answered with yes. |
|  |  |  |
| Other | Is the word 'validation', 'validated', 'characterization', 'specificity', 'reliability' mentioned with regard to antibodies in the paper/SI? |  |
|  | Comments: | Any unusual things or doubts about the paper |
|  | Elaborate explanation validation: | Copy and paste sections of paper/SI describing validation to illustrate. |

## Part 2. Questions answered if at least one antibody was validated

| Category | Question about article | Further explanation |
| --- | --- | --- |
| Validation | Does the article show proof of validation or explain validation? | Experimental proof or reference to where to find proof. E.g. in case the article only shows a statement such as: ‘all antibodies were validated,’ this question is answered with no. |
|  | How many of the used antibodies are validated? | Vague statements like ‘all antibodies were validated’ get benefit of the doubt. |
|  | Where is the antibody validation information mentioned? SI or main text? |  |
|  | Does the article mention validation of some antibodies but not of others? |  |
|  | Does the article mention different types of validation for different antibodies used? |  |
|  | Does the article mention multiple types of validation per antibody? |  |
|  | Are all primary antibodies validated? |  |
|  |  |  |
| Type of antibody validation | Is a reference to validation by the antibody supplier given? | Statements, such as: ‘the specificity of the antibody has been assessed by the manufacturer of the antibody,’ mean this question is answered with yes. |
|  | Is a reference to antibody validation in the literature given? | Statements, such as: ‘the specificity of the antibody was previously established by WB’, followed with a citation to the literature mean this question is answered with yes.  (In this case the question about the type of validation by researchers themselves ‘is the antibody validated by using WB?’ is answered with no because validation was not carried out by the researchers themselves.) |
|  | Is a reference to antibody validation information from a database given? | Statements, such as: ‘the specificity of the antibody was previously established by knockout experiments,’ followed with a citation to a database mean this question is answered with yes. |
|  | Is antibody validation carried out by the authors of the article (by researchers themselves)? | Any type of validation that was carried out by researchers themselves, even if validation was not the purpose of researchers. E.g. picture of WB showing knockout and wild type samples stained with antibody results in a yes. |

## Part 3. Questions answered about validation by researchers

| Category | Question about article | Further explanation |
| --- | --- | --- |
| Type of validation by researchers themselves | Is the method molecular weight similar to that of target (in WB) used? | Statements, such as: ‘as expected for this protein, bands were detected at 33 kDa,’ result in a yes. |
|  | Is the method spatial localization (similar staining patterns literature/ supplier/ database) used? | Statements, such as: ’the immunostaining pattern was similar to patterns previously found by another research group,’ result in a yes. |
|  | Is the method pre-adsorption/ blocking peptide used? | Statements, such as: ‘staining was abolished following pre-adsorption’ result in a yes. |
|  | Is a secondary antibody used without a primary one? | Statements, such as: ‘no staining was observed when omitting the primary antibody,’ result in a yes. |
|  | Is in situ hybridization used?^[[1]](#footnote-1)^ | Antibody validation does not have to be explicitly mentioned. E.g. if the ISH mRNA pattern of one figure matches the IHC pattern of another figure stained with antibody against same protein. |
|  | Are other RNA based methods used? ^1^ | E.g. RNA expression level of protein matches that of protein visualized with antibody. |
|  | Are the following methods used?  Protein array?^1^  Tissue microarray (TMA)?^1^  Epitope mapping?^1^  Affinity measurement?^1^ | For an elaborate explanation of these methods see Appendix 2. |
|  |  |  |
| Five Pillars | Is five pillar validation used? | If answered with yes, questions were answered about the type of five pillar validation. |
| Type five pillar validation | Is the type genetic strategies used? | E.g. picture of WB showing staining in wildtype cells but no staining in cells were protein expression was silenced. |
|  | Is the type orthogonal strategies used? | E.g. similar pattern with antibody method and antibody independent method. |
|  | Is the type independent antibody strategies used? | Two antibodies against same protein give similar results. |
|  | Is the type expression of tagged protein used? | E.g. tagged protein stained with antibody against tag and with antibody against protein. |
|  | Is the type immunocapture-MS used? | E.g. after immunoaffinity purification, proteins were identified with mass spectrometry. |
|  |  |  |
| \| Positive control \| Was a positive control used? \| If answered with yes, questions were answered about the type of positive control that was used. \| \| --- \| --- \| --- \| \| Type of positive control \| Was a purified target or recombinant protein used? \|  \| \| Were transfected or transgenic samples used? \| E.g. expression of tagged protein only detected in samples from mice that were treated with plasmid containing protein’s DNA and not in control mice. \| \| Was a sample known to express the target used? \| E.g. tagged protein used in reaction, and then reaction mixture was analyzed with WB. \| \| Was another positive control method used? \|  \| \|  \|  \|  \| \| Negative control \| Was a negative control used? \| If answered with yes, questions were answered about the type of negative control that was used. \| \| Type of negative control \| Was a knockout sample used? \|  \| \| Was siRNA/blocking RNA used? \|  \| \| Were non-transgenic or non-transfected samples used? \| E.g. tagged protein is expressed and antibody is used to detect this protein, but antibody is also used in wild type sample, which then counts as a negative control. \| \| Is a sample, which is known not to express the target, used? \| E.g. no staining of samples where chemical reaction was used to cleave off part of the protein antibodies bind to. \| \| Was another negative control method used? \|  \| \|  \|  \|  \| \| Other \| Was another validation method used? Which one? \|  \| | | |

# 3 - Methods of antibody validation

This section will shortly describe the different kinds of validation that can be carried out by researchers themselves, as have been proposed in the literature.

1. Frequently used validation methods

#### Pre-adsorption/blocking peptides (in IHC)

A method of validation that is often mentioned is pre-adsorption, used in IHC. In this process, the antibody is incubated in great excess with the antigen (e.g. peptide sequence) it was raised against. It is then ‘blocked’ by this peptide and cannot bind to other peptides in the sample of interest anymore. After incubation, antibodies with and without this blocking peptide are used to stain tissue that is known to express the target. If the blocked peptide does not show any staining of the tissue, the antibody is specific for the target peptide from which it was generated. However, this does not necessarily mean that the antibody cannot still be cross-reactive with different proteins present in the sample. If the blocked antibody cannot bind to the target protein in a sample, it can also not bind to off-target proteins in that sample. Hence pre-adsorption can be used to show that an antibody is wrong (if it still stains tissue after addition of the blocking peptide), but it cannot show that an antibody is right (that it is not cross-reactive) [3, 4]. Several antibodies that had been validated using this method proved to actually be non-selective upon more thorough validation [3].

Pre-adsorption can be performed with the (synthetic) antigen against which the antibody was generated or with, for example, purified target protein. This makes a difference in how the validation of the antibody should be evaluated: that an antibody binds to the synthetic antigen it was generated with does not necessarily mean it also binds to the target protein in its native conformation.

#### Omission of primary antibody

Leaving the primary antibody out of the experiment and only using the secondary antibody can be useful when there is a high background staining to differentiate between the background staining and the antibody-protein specific staining [5]. This method is also used by researchers to tell something about the specificity of the primary antibody. When the primary antibody is omitted, no staining should be observed. This method cannot determine whether the antibody is cross-reactive.

However, it is disputed whether this counts as evidence for the specificity of the primary antibody. Using a secondary antibody without the primary antibody reveals something about the specificity of the secondary antibody, not the primary one; it is a control only for the staining method [6, 7].

Another, related, method that can be used is leaving the primary antibody out of the experiment and adding a control antibody that binds to a completely different, unrelated protein but is of the same isotype as the primary antibody [7]. Pre-immune serum, derived from the same animal before immunization, could also be used as a control [5]. The control antibody should not bind to the target protein, but it likely does bind to the secondary antibody. However, since it should not bind to the target no staining should be observed.

#### Spatial localization/similar staining patterns

Spatial localization is the comparison of the distribution of the antibody signal in cells, subcellular compartments, or tissue with the existing scientific literature on the location of the protein target [8]. If the protein is well-known or has been partly characterized before, information about the protein and its localization in cells and tissues can be found in online databases or scientific publications. Observing similar staining patterns with the antibody as have been described in the scientific literature, by a supplier or by an antibody database, is then a sign that the antibody used is specific for that protein [9].

#### Molecular weight markers (in WB)

In WB, molecular weight markers can be used to show if the antibody stains a protein of the correct molecular weight [10]. The staining at unexpected molecular weight or staining of multiple bands could be a first indication that the antibody is not specific for the protein [3]. Staining of a band of the correct molecular weight suggests that the antibody binds to its target protein. However, it could also be the case that it binds to proteins of similar molecular weight [9].

1. Positive and negative controls

#### Positive control

Another method of validation is to stain the purified or recombinant protein with the antibody. If it is stained, this indicates that the antibody binds to its target [11]. However, purified protein is not the same as the complex biological sample that the antibody would normally be used in. A physiologically relevant control sample that is known to (over)express the protein of interest would be better to use [12]. Cells that are not expressing the target protein, but have been transfected with the protein of interest, can also be used as controls [3].

Overexpression of the target should be done with caution, since overexpressed systems are prone to abnormal results in which the target can be mislocalized [8]. This method can thus be useful to determine if the antibody binds to the target, but should be applied with caution if the aim is to visualize the location of protein expression in the cell.

These methods would serve as positive controls and only using a positive control is not sufficient to ensure antibody reliability, since this control does not show whether it stains other proteins also present in the sample [13]. As with pre-adsorption, this method can show that the antibody binds to the target, but not that it is not cross-reactive.

#### Negative control

Negative controls are tissues or cells that do not contain the target protein. In WB they could be samples obtained from knockout animals, that do not express the target protein [13]. Cells in which the protein expression is stopped by RNA interference can also be used [14]. Cells or tissues which are known not to express the target protein can also be appropriate negative controls [3]. If the antibody stains these samples, then cross-reactivity is shown, and the antibody is not reliable for the target protein.

1. Five pillars of antibody validation

In 2016, five pillars of antibody validation, to be used by researchers as well as producers of antibodies, have been proposed by Uhlén et al. The five pillars provide strategies to test if an antibody is specific to its target and to show if there is cross-reactivity or not in the application tested. Uhlén et al. state that at least one of the five strategies should be used to claim that an antibody has been validated for use under specific conditions [4]. The five pillars are described below.

#### Genetic strategies

This approach is similar to that of negative controls described above. The specificity of the antibody is tested in control samples in which the target (e.g. protein) is not expressed. This can be accomplished by knocking out the gene with CRISPR-Cas9 or silencing it with RNA interference to reduce or completely stop the protein expression. If a signal is observed in these negative controls, the antibody is cross-reactive and not specific for only its target protein. A drawback to this approach is that it cannot be used for human tissue samples and body fluids [4].

#### Orthogonal strategies

With this strategy, an antibody-independent method is used to determine the amount of target protein present across many samples. Then the antibody is used to determine this again. This should be done for a set of samples with varying protein expression. If both methods then correlate strongly in their outcomes the antibody is specific for its target [4]. In situ hybridization, as well as other methods that compare RNA expression to protein expression, can be seen as orthogonal strategies.

#### Independent antibody strategies

Two or more independent antibodies for the same target are used to bind to this target in more than one assay. If all antibodies stain the same bands (in WB) or tissue (in IHC), these antibodies are found to be specific for that target [4]. This method should be used with caution since multiple suppliers sometimes sell the same antibody from the same original manufacturer under different names. Researchers then think they are using different antibodies while they are actually using the exact same one. It is therefore important to choose antibodies that bind to different epitopes of the target [5]. This is also important to minimize the chances that all antibodies are binding to the same region of an non-target protein [4].

#### Expression of tagged proteins

The protein of interest is expressed with an affinity tag or a fluorescent protein attached to it. This allows for detection of the protein without the antibody of interest. This detection can then be compared with the detection of the protein with the antibody. If this pattern correlates strongly, the antibody is specific for that target protein. A drawback of this approach is that the attachment of such a tag could change protein activity [4].

#### Immunocapture followed by mass spectrometry (MS)

Immunocapture captures the target protein from a solution by binding it to the antibody. The proteins that are isolated in this way can then be identified by MS analysis. In this way, all different proteins that bind to the antibody can be identified. An antibody is considered to be specific if the top three peptides that are identified are derived from the target protein. A drawback is that it is difficult to distinguish between proteins that interact with the antibody and proteins that interact with the target protein but not with the antibody [4].

1. Other methods of validation

#### Protein/peptide array

A protein or peptide array can be used to determine the specific, versus cross-reactive, binding of an antibody [4]. This array consists of many different peptides or proteins displayed on the surface of a chip. This chip can then be incubated with the antibody, after which the antigen-antibody binding can be visualized by a labeled secondary antibody. A drawback of this method is that it is not possible to determine with certainty that an antibody is not cross-reactive since not all proteins present in a biological sample will be represented on the protein array.

#### RNA-based methods:

There is a strong relationship between the mRNA levels expressed and the protein levels expressed in a cell. Comparing mRNA expression to protein expression can therefore be a method to confirm antibody specificity. RNA expression does not prove protein expression, amongst others, since proteins can also be transported to other sites away from the site where mRNA is expressed [9]. However, researchers can use the correlation as an easy method to check the specificity of the antibody. RNA levels that differ greatly from antibody-binding levels could be an early warning sign that something is wrong with the antibody binding [15]. Apart from the fact that there is sometimes no correlation between RNA and protein expression, another drawback of this method is that RNA expression cannot be used to determine the specificity of antibodies used against post-translationally modified proteins.

Some RNA methods that can be used to determine antibody specificity:

#### Single-cell RT-PCR/comparison of RNA-Seq data

Single-cell RT-PCR and comparison with online RNA sequencing data are quantitative methods used to determine if the protein expression levels match the mRNA expression levels [9].

#### In situ hybridization (ISH)

In situ hybridization is a qualitative method used to visualize RNA expression in cells or tissues. It uses labeled RNA probes to do this. It can be used to determine if the protein distribution matches that of the RNA in cells or tissue [9]. If the IHC staining pattern matches that of in situ hybridization (ISH) of mRNA of the same protein, the antibody is likely specific for that protein.

#### Tissue microarray (TMA)

Tissue microarrays contain up to 1,000 different tissue sample pellets on a single microscope slide, so that they can be evaluated simultaneously [16]. TMAs can be used to evaluate antibody binding across a multitude of samples that differ in the expression of the target protein [8]. A good antibody will only stain the pellets that contain the target, the staining will decrease with decreasing antibody dilutions and the staining will be similar to staining reported in the literature [3]. A side note to this method could be that it might be better to carefully select a positive and negative control sample with and without the target protein instead of using 1,000 random tissue samples that have not been selected specifically as controls for the target.

#### Epitope mapping

Epitope mapping can consist of several methods, such as peptide arrays and alanine-scanning, which are used to determine the specific epitope an antibody binds to [4].

#### Affinity measurements

The binding affinity of an antibody to its antigen can be measured. A surface plasmon resonance immunoassay could be a method to determine the binding parameters of the antibody to its antigen [4].

# 4 - Coding examples

| Box 1. Examples of validation with genetic strategies and how they were coded |
| --- |
| Tags Recombinant proteins, engineered to express small peptide tags, can be introduced into cells where they are not naturally occurring, for instance by using an expression vector that introduces the DNA of the recombinant protein into the cell. This can be done for many different reasons, such as to detect or isolate a target protein in the cell. The HA-tag, Myc-tag, FLAG-tag or GFP-tag are frequently used in combination with antibodies against these tags to detect a protein of interest. When these tags are used, often samples with and without these tags are stained with the anti-tag antibody, which means that these antibodies are validated by genetic strategies according to our validation criteria. This means that these antibodies were not validated with the purpose to validate them but as a consequence of the experimental procedures that were used. Anti-HA, anti-Myc, anti-FLAG or anti-GFP was used in 37, and marked as validated in 25, of the 120 articles in the sample.  An example from one of the articles in the sample is the detection of the Myc tag with the anti-Myc antibody. Cells transduced with a Myc-containing vector show staining with anti-Myc, while untreated cells do not show any staining [1]. The anti-Myc antibody is now validated with a positive control (cells in which Myc was introduced) and negative control (untreated cells in which Myc is not present). Furthermore, this type of validation fits into the category of validation with genetic strategies of the five pillars of validation.  Figure from [1] |
| 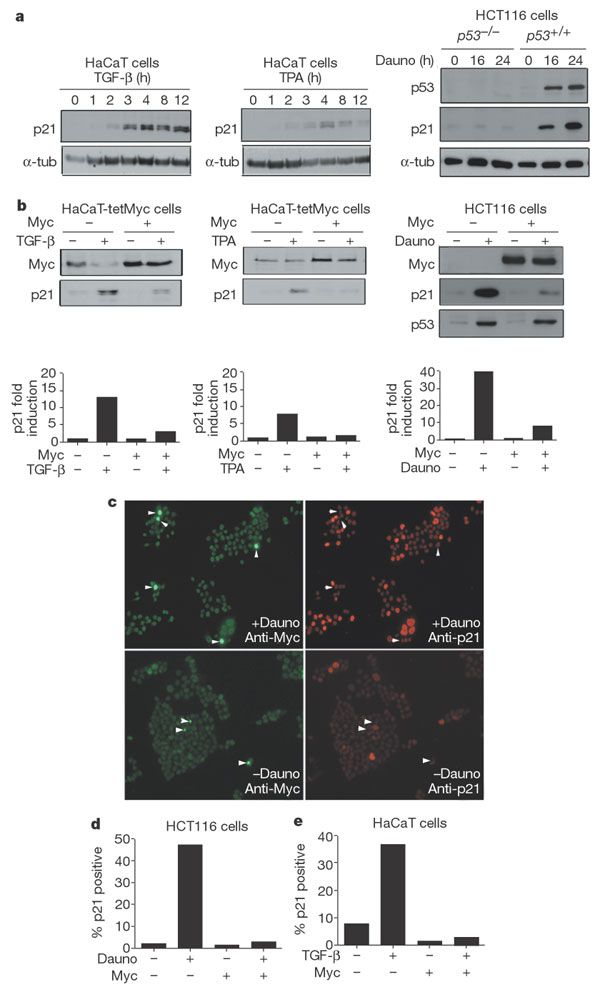Other examples - Knockout Figure from [1]  p53^-/-^ cells show no staining with an antibody against tumor suppressor protein p53, while p53^+/+^ cells do. This shows that the antibody specifically stains samples in which p53 is present [1]. This type of validation was marked as validation by negative control (knockout) and by genetic strategies of the five pillars. |
| Other examples - RNA interference Atg7 defective cells were generated by inhibiting gene expression of the Atg7 gene with RNA interference. The cells in which Atg7 was silenced show no staining with anti-ATG7, while wild type cells do [2]. This type of validation was marked as validation by negative control (RNA interference) and by genetic strategies of the five pillars.  Figure from [2] |

# References

1. Seoane, J., H.-V. Le, and J. Massagué, *Myc suppression of the p21Cip1 Cdk inhibitor influences the outcome of the p53 response to DNA damage.* Nature, 2002. **419**: p. 729.

2. Abu-Remaileh, M., et al., *Lysosomal metabolomics reveals V-ATPase- and mTOR-dependent regulation of amino acid efflux from lysosomes.* Science, 2017. **358**(6364): p. 807.

3. Bordeaux, J., et al., *Antibody validation.* Biotechniques, 2010. **48**(3): p. 197.

4. Uhlen, M., et al., *A proposal for validation of antibodies.* Nature Methods, 2016. **13**: p. 823.

5. Ivell, R., K. Teerds, and G.E. Hoffman, *Proper Application of Antibodies for Immunohistochemical Detection: Antibody Crimes and How to Prevent Them.* Endocrinology, 2014. **155**(3): p. 676-687.

6. Hewitt, S.M., et al., *Controls for Immunohistochemistry: The Histochemical Society’s Standards of Practice for Validation of Immunohistochemical Assays.* Journal of Histochemistry and Cytochemistry, 2014. **62**(10): p. 693-697.

7. Howat, W.J., et al., *Antibody validation of immunohistochemistry for biomarker discovery: Recommendations of a consortium of academic and pharmaceutical based histopathology researchers.* Methods, 2014. **70**(1): p. 34-38.

8. GBSI, *Drivers for Adoption: Ensuring Acceptance and Compliance by the Research Community*. 2016, Global Biological Standards Institute.

9. O'hurley, G., et al., *Garbage in, garbage out: a critical evaluation of strategies used for validation of immunohistochemical biomarkers.* Molecular oncology, 2014. **8**(4): p. 783-798.

10. Saper, C.B. and P.E. Sawchenko, *Magic peptides, magic antibodies: guidelines for appropriate controls for immunohistochemistry.* Journal of Comparative Neurology, 2003. **465**(2): p. 161-163.

11. Voskuil, J.L.A., *Commercial antibodies and their validation.* F1000Research, 2014. **3**: p. 232.

12. Acharya, P., A. Quinlan, and V. Neumeister, *The ABCs of finding a good antibody: How to find a good antibody, validate it, and publish meaningful data.* F1000Research, 2017. **6**.

13. Gilda, J., et al., *Western Blotting Inaccuracies with Unverified Antibodies: Need for a Western Blotting Minimal Reporting Standard (WBMRS)*. Vol. 10. 2015. e0135392.

14. Weller, M.G., *Quality issues of research antibodies.* Analytical chemistry insights, 2016. **11**: p. ACI. S31614.

15. Dove, A., *Technology Feature| Agreeable antibodies: Antibody validation challenges and solutions.* Science, 2017. **357**(6356): p. 1165-1167.

16. Ramos-Vara, J. and M. Miller, *When tissue antigens and antibodies get along: revisiting the technical aspects of immunohistochemistry—the red, brown, and blue technique.* Veterinary pathology, 2014. **51**(1): p. 42-87.

1. These methods were combined with the category ‘Other’ during data analysis because they were not used prevalently enough to consider separately. [↑](#footnote-ref-1)
