# Supplementary material for: The effect of journal guidelines on the reporting of antibody validation
Source: PeerJ. 2020 Jun 3;8:e9300. doi: 10.7717/peerj.9300 (PMC7275675; doi:10.7717/peerj.9300)
Supplement: Article S4 [file peerj-08-9300-s004.docx]

**Data dictionary**

Hoek, J.M., Hepkema, W.M., Halffman, W. (2020) Can journal guidelines improve the reporting of antibody validation?

**Files:**

- **Antibody validation information data set:**

This dataset shows antibody information extracted by author JMH from 120 publications in the journals *Nature, Science, Journal of Comparative Neurology* and *Neuroscience* between August 2002 and August 2003 or June 2017 and June 2018. In the data set, the value 1 means yes, 0 means no, 9 means not applicable, 999 means not clear.

- **Interrater reliability data set:**

This dataset contains a random sample of articles from the ‘antibody validation information data set’ that were evaluated by both author JMH and WMH. This table displays both responses next to each other. Questions were answered with y (yes), n (no), na (not applicable) or ? (not clear).

- **R code for the analysis:**

This file contains the R code that was used for the analysis of both the ‘antibody validation information data set’ and the ‘interrater reliability data set’.

- **Coding protocol:**

This document contains information on how the antibody information was extracted from the evaluated articles. It contains a more elaborate explanation of the antibody validation table that we used to extract the antibody information from the articles. It also contains an overview of different methods of antibody validation and examples that illustrate how we approached the extraction of antibody validation and coding in the table. This protocol should be used supplementary to our data sets to understand how we obtained our data and what we mean with the different variables. It can also be used in a possible replication of our research. Both raters (JMH and WMH) used this protocol as guidance during the extraction of antibody information from the publications.

**Variables:**

Short explanation of the headers in the ‘antibody validation information data set’ (for a more elaborate explanation see the ‘coding protocol’).

| **Name** | **Meaning** | **Values** |
| --- | --- | --- |
| Journal | Which journal is the article from? | JCN, Nature, Neuroscience or Science |
| Journalnr | Gives a number to the journals | JCN=1, Nature=2, Neuroscience=3, Science=4 |
| with_guidelines | Does the journal have antibody guidelines or not? | With guidelines=1, without guidelines=0 |
| Times_cited | How often was the publication cited at the date of retrieval according to Web of Science? | Number |
| Date_published | Publication date of article, obtained from Web of Science | Day-month-year |
| After_guidelines | Was the article published before or after the introduction of guidelines by two of the journals? | Before introduction of guidelines =0, after=1 |
| Date_received | At what date was the article submitted according to Web of Science | Day-month-year |
| Section_devoted_to_abs | Is there a section devoted to antibodies in the paper? | Yes=1, no=0, not applicable=9, not clear =999 |
| How_many_different_abs_used | How many different antibodies were used in the paper? | Number, not clear=999 |
| Application_using_abs | In which application were the antibodies used? Multiple answers possible. | Abbreviation of applications (for explanation of the applications see ‘coding protocol’) |
| Commercial_abs | Were commercial antibodies used? | Yes=1, no=0, not applicable=9, not clear =999 |
| Abs_byresearchers | Are antibodies used that are generated by the researchers themselves or obtained from other researchers? | Yes=1, no=0, not applicable=9, not clear =999 |
| Ab_info_present | Is any antibody information (characteristics) present? | Yes=1, no=0, not applicable=9, not clear =999 |
| Basic_ab_info_complete_commercial | Is the basic antibody information of all commercial antibodies complete? | Yes=1, no=0, not applicable=9, not clear =999 |
| Basic_ab_info_complete_noncommercial | Is the basic antibody information of all non-commercial antibodies complete? | Yes=1, no=0, not applicable=9, not clear =999 |
| Basic_info_all_primary_abs_complete | Is the basic antibody information of all primary antibodies complete? | Yes=1, no=0, not applicable=9, not clear =999 |
| Basic_info_all_primary_abs_complete_na_no | Is the basic antibody information of all primary antibodies complete? The answer not applicable was coded as no here. | Yes=1, no=0, not clear =999 |
| Validation_info_present | Is any validation information about any of the antibodies present? | Yes=1, no=0, not applicable=9, not clear =999 |
| Proof_validation_present | Does the article show proof of validation or explain validation? | Yes=1, no=0, not applicable=9, not clear =999 |
| How_many_abs_validated | How many of the used antibodies are validated? | Number, not clear =999 |
| Percentage_abs_validated | What percentage of antibodies in the paper is validated? Calculated from How_many_different_abs_used and How_many_abs_validated | Percentage, not clear = 999 |
| Place_info_validation | Where is the antibody validation information mentioned? SI or main text? | SI and/or main, not applicable=9, not clear=999 |
| Validation_some_abs_not_others | Does the article mention validation of some antibodies but not of others? | Yes=1, no=0, not applicable=9, not clear =999 |
| Different_types_validation_different_abs | Does the article mention different types of validation for different antibodies used? | Yes=1, no=0, not applicable=9, not clear =999 |
| Multiple_types_validation_per_ab | Does the article mention multiple types of validation per antibody? | Yes=1, no=0, not applicable=9, not clear =999 |
| All_primary_ab_validated | Are all primary antibodies validated? | Yes=1, no=0, not applicable=9, not clear =999 |
| All_primary_ab_validated_na_no | Are all primary antibodies validated? The answer not applicable was coded as no here. | Yes=1, no=0, not clear =999 |
| Reference_validation_supplier | Is a reference to validation by the antibody supplier given? | Yes=1, no=0, not applicable=9, not clear =999 |
| Reference_validation_literature | Is a reference to antibody validation in the literature given? | Yes=1, no=0, not applicable=9, not clear =999 |
| Reference_validation_database | Is a reference to antibody validation information from a database given? | Yes=1, no=0, not applicable=9, not clear =999 |
| Validation_by_researcher | Is antibody validation carried out by the authors of the article (by researchers themselves)? | Yes=1, no=0, not applicable=9, not clear =999 |
| MW_WB | Is the method molecular weight similar to that of target (in WB) used? | Yes=1, no=0, not applicable=9, not clear =999 |
| Spatial_localization | Is the method spatial localization (similar staining patterns literature/ supplier/ database) used? | Yes=1, no=0, not applicable=9, not clear =999 |
| Preabsorption | Is the method pre-adsorption/ blocking peptide used? | Yes=1, no=0, not applicable=9, not clear =999 |
| Secondary_without_primary | Is a secondary antibody used without a primary one? | Yes=1, no=0, not applicable=9, not clear =999 |
| Protein_array | Is the method protein array used?^1^ | Yes=1, no=0, not applicable=9, not clear =999 |
| ISH | Is in situ hybridization used?^[[1]](#footnote-1)^ | Yes=1, no=0, not applicable=9, not clear =999 |
| Other_RNA_method | Are other RNA based methods used? ^1^ | Yes=1, no=0, not applicable=9, not clear =999 |
| Tissuemicroarray | Is the method Tissue microarray (TMA) used?^1^ | Yes=1, no=0, not applicable=9, not clear =999 |
| Epitopemapping | Is the method Epitope mapping used?^1^ | Yes=1, no=0, not applicable=9, not clear =999 |
| Affinitymeasurement | Is the method affinity measurement used?^1^ | Yes=1, no=0, not applicable=9, not clear =999 |
| Five_pillars | Is five pillar validation used? | Yes=1, no=0, not applicable=9, not clear =999 |
| Genetic | Of the five pillars, is the type genetic strategies used? | Yes=1, no=0, not applicable=9, not clear =999 |
| Orthogonal | Of the five pillars, is the type orthogonal strategies used? | Yes=1, no=0, not applicable=9, not clear =999 |
| Independent_ab | Of the five pillars, is the type independent antibody strategies used? | Yes=1, no=0, not applicable=9, not clear =999 |
| Expression_tagged_protein | Of the five pillars, is the type expression of tagged protein used? | Yes=1, no=0, not applicable=9, not clear =999 |
| Immunocapture_MS | Of the five pillars, is the type immunocapture followed by Mass Spectrometry used? | Yes=1, no=0, not applicable=9, not clear =999 |
| Positive_control | Was a positive control used? | Yes=1, no=0, not applicable=9, not clear =999 |
| Purified_target_recombinant_protein | Of the types of positive control, was a purified target or recombinant protein used? | Yes=1, no=0, not applicable=9, not clear =999 |
| Transfected | Of the types of positive control, were transfected or transgenic samples used? | Yes=1, no=0, not applicable=9, not clear =999 |
| Known_to_express_target | Of the types of positive control, was a sample known to express the target used? | Yes=1, no=0, not applicable=9, not clear =999 |
| Other_positive_control | Was another positive control method used? | Yes=1, no=0, not applicable=9, not clear =999 |
| Negative_control | Was a negative control used? | Yes=1, no=0, not applicable=9, not clear =999 |
| KO | Of the types of negative control, was a knockout sample used? | Yes=1, no=0, not applicable=9, not clear =999 |
| siRNA_blocking_RNA | Of the types of negative control, was siRNA/blocking RNA used? | Yes=1, no=0, not applicable=9, not clear =999 |
| Non_transgenic_transfected | Of the types of negative control, were non-transgenic or non-transfected samples used? | Yes=1, no=0, not applicable=9, not clear =999 |
| Known_not_to_express_target | Of the types of negative control, was a sample, which is known not to express the target, used? | Yes=1, no=0, not applicable=9, not clear =999 |
| Other_negative_control | Was another negative control method used? | Yes=1, no=0, not applicable=9, not clear =999 |
| Other_validation_method | Was another validation method used? Which one? | y or explanation of method = yes, n=no, na=not applicable |
| Othernr | Was another validation method used? Gives the other validation methods a number. | Yes=1, no=0, not applicable=9, not clear =999 |
| Othercomplete | Addition of all ‘other’ methods. Combines Protein_array, ISH, Other_RNA_method, Tissuemicroarray, Epitopemapping, Affinitymeasurement and Othernr. | Yes=1, no=0, not applicable=na, not clear =999 |
| Word_validation_mentioned | Is the word 'validation', 'validated', 'characterization', 'specificity', 'reliability' mentioned with regard to antibodies in the paper/SI? | Yes=1, no=0, not applicable=9, not clear =999 |
| Comments | Any unusual things or doubts about the paper. | Comments |
| Elaborate_explanation | Elaborate explanation of the antibody (validation) information found in the evaluated article. | Copy pasted pieces from evaluated article where antibody information was found |

1. These methods were combined with the category ‘Other’ during data analysis because they were not used prevalently enough to consider separately. [↑](#footnote-ref-1)
