# Supplementary material for: The effect of journal guidelines on the reporting of antibody validation
Source: PeerJ. 2020 Jun 3;8:e9300. doi: 10.7717/peerj.9300 (PMC7275675; doi:10.7717/peerj.9300)
Supplement: Supplemental Information 1 [file peerj-08-9300-s007.docx]

| ***Table S1.* Percentage of validated antibodies, number of validated antibodies, and number of used antibodies per article for each journal.** | | | | | | | |
| --- | --- | --- | --- | --- | --- | --- | --- |
| ***JCN*** | | ***Neuroscience*** | | ***Nature*** | | ***Science*** | |
| **< Aug 2003** | **> June 2017** | **< Aug 2003** | **> June 2017** | **< Aug 2003** | **> June 2017** | **< Aug 2003** | **> June 2017** |
| 0% (0/3) | 0% (0/2) | 0% (0/1) | 0% (0/2) | 0% (0/1) | 0% (0/1) | 0% (0/2) | 0% (0/3) |
| 0% (0/8) | 17% (1/6) | 0% (0/3) | 0% (0/2) | 0% (0/2) | 0% (0/9) | 0% (0/3) | 0% (0/4) |
| 0% (0/9) | 25% (1/4) | 0% (0/3) | 0% (0/3) | 0% (0/7) | 19% (4/21) | 0% (0/4) | 0% (0/11) |
| 0% (0/10) | 25% (1/4) | 0% (0/3) | 0% (0/4) | 14% (2/14) | 25% (7/28) | 0% (0/6) | 0% (0/11) |
| 14% (1/7) | 47% (9/19) | 0% (0/9) | 0% (0/4) | 15% (2/13) | 27% (6/22) | 0% (0/7) | 0% (0/20) |
| 25% (3/12) | 50% (6/12) | 0% (0/9) | 0% (0/5) | 17% (3/18) | 33% (3/9) | 0% (0/10) | 9% (1/11) |
| 33% (1/3) | 63% (5/8) | 0% (0/10) | 0% (0/6) | 18% (2/11) | 50% (1/2) | 10% (1/10) | 10% (1/10) |
| 40% (2/5) | 64% (7/11) | 0% (0/12) | 0% (0/6) | 20% (1/5) | 60% (3/5) | 10% (1/10) | 10% (3/29) |
| 43% (3/7) | 67% (2/3) | 11% (1/9) | 0% (0/13) | 25% (1/4) | 75% (9/12) | 17% (1/6) | 14% (2/14) |
| 63% (5/8) | 80% (4/5) | 50% (2/4) | 13% (1/8) | 33% (1/3) | 86% (25/29) | 26% (5/19) | 17% (1/6) |
| 100% (1/1) | 83% (5/6) | 60% (3/5) | 13% (1/8) | 33% (4/12) | 100% (1/1) | 33% (1/3) | 20% (1/5) |
| 100% (4/4) | 100% (5/5) | 75% (3/4) | 25% (1/4) | 45% (5/11) | 100% (1/1) | 33% (2/6) | 30% (3/10) |
| 100% (4/4) | 100% (10/10) | 100% (2/2) | 33% (1/3) | 58% (7/12) | 100% (4/4) | 43% (6/14) | 38% (6/16) |
| 100% (7/7) | 100% (12/12) | 100% (5/5) | 33% (4/12) | 67% (2/3) | 100% (5/5) | 50% (1/2) | 56% (10/18) |
| 100% (9/9) | NA | NA | 40% (2/5) | 100% (2/2) | NA | 50% (1/2) | 58% (7/12) |

Can Journal Guidelines Improve the Reporting of Antibody Validation? - Supplemental Tables

# Validation information – at least one antibody

| ***Table S2.* Difference in percentage of articles reporting validation of at least one antibody per article before and after introduction of guidelines.^1^** | | | | | |
| --- | --- | --- | --- | --- | --- |
|  | **< Aug 2003**  ***n/N* (%)** | **> June 2017**  ***n/N* (%)** | ***P*-value** | **Adjusted *P-*value** | **Odds ratio (95% CI)** |
| *JCN* | 11/15 (73%) | 14/15 (93%) | 0.1648 | 1.0000 | 4.837 (0.539 – INF) |
| *Nature* | 12/15 (80%) | 13/15 (87%) | 0.5000 | 1.0000 | 1.599 (0.212 – INF) |
| Total with guidelines | 23/30 (77%) | 27/30 (90%) | 0.1495 | 1.0000 | 2.694 (0.665 – INF) |
| *Neuroscience* | 7/15 (47%) | 6/15 (40%) | 1.0000^2^ | 1.0000^2^ | 0.769 (0.141 – 4.052) |
| *Science* | 9/15 (60%) | 10/15 (67%) | 1.0000^2^ | 1.0000^2^ | 1.321 (0.237 – 7.671) |
| Total without guidelines | 16/30 (53%) | 16/30 (53%) | 1.0000^2^ | 1.0000^2^ | 1.000 (0.322 – 3.104) |
| Total all journals | 39/60 (65%) | 43/60 (72%) | 0.5564^2^ | 1.0000^2^ | 1.358 (0.587 – 3.180) |
| ^1^*P-*values indicate the significance of the difference in proportion between samples before (before August 2003) and after (after June 2017) *JCN* and *Nature* introduced guidelines. *P*-values were determined with Fisher’s exact test (one-tailed, unless stated otherwise). Adjusted *P*-values were determined using the Holm-Bonferroni method.  ^2^ Two-tailed. | | | | | |

| ***Table S3.* Differences in percentage of articles reporting validation of at least one antibody per article between similar journals with and without guidelines. ^1^** | | | | | |
| --- | --- | --- | --- | --- | --- |
|  | ***n/N* (%)** | ***n/N* (%)** | ***P*-value** | **Adjusted *P-value*** | **Odds ratio (95% CI)** |
|  | ***JCN*** | ***Neuroscience*** |  |  |  |
| < Aug 2003 | 11/15 (73%) | 7/15 (47%) | 0.2635^2^ | 1.0000^2^ | 3.020 (0.547 – 19.536) |
| > June 2017 | 14/15 (93%) | 6/15 (40%) | 0.0026 | 0.1325 | 18.722 (2.453 – INF) |
|  | ***Nature*** | ***Science*** |  |  |  |
| < August 2003 | 12/15 (80%) | 9/15 (60%) | 0.4270^2^ | 1.0000^2^ | 2.579 (0.411 – 20.449) |
| > June 2017 | 13/15 (87%) | 10/15 (67%) | 0.1949 | 1.0000 | 3.125 (0.525 – INF) |
|  | **Total with guidelines** | **Total without guidelines** |  |  |  |
| < August 2003 | 23/30 (77%) | 16/30 (53%) | 0.1033^2^ | 1.0000^2^ | 2.823 (0.839 – 10.305) |
| > June 2017 | 27/30 (90%) | 16/30 (53%) | 0.0017 | 0.0907 | 7.597 (2.107 – INF) |
|  | | | |  |  |
| ^1^*P-*values indicate the significance of the difference in proportion between samples of similar journals (*JCN* and Neuroscience; *Nature* and Science) with (*JCN* and Nature) or without (*Neuroscience* and *Science*) guidelines. *P*-values were determined with Fisher’s exact test (one-tailed, unless stated otherwise). Adjusted P-values were determined using the Holm-Bonferroni method.  ^2^ Two-tailed. | | | | | |

# All primary antibodies validated

| ***Table S4.* Difference in percentage of articles that reports validation of all primary antibodies used before and after introduction of guidelines.^1^** | | | | | |
| --- | --- | --- | --- | --- | --- |
|  | **< Aug 2003**  *n/N* (%) | **> June 2017**  *n/N* (%) | ***P*-value** | **Adjusted *P-value*** | **Odds ratio (95% CI)** |
| *JCN* | 6/15 (40%) | 9/14 (64%) | 0.1749 | 1.0000 | 2.605 (0.602 – INF) |
| *Nature* | 1/15 (7%) | 4/14 (29%) | 0.1433 | 1.0000 | 5.289 (0.585 – INF) |
| Total with guidelines | 7/30 (23%) | 13/28 (46%) | 0.0575 | 1.0000 | 2.795 (0.963 – INF) |
| *Neuroscience* | 3/15 (20%) | 0/15 (0%) | 0.2241^2^ | 1.0000^2^ | 0.000 (0.000 – 2.316) |
| *Science* | 1/15 (7%) | 0/15 (0%) | 1.000^2^ | 1.0000^2^ | 0.000 (0.000 – 39.001) |
| Total without guidelines | 4/30 (13%) | 0/30 (0%) | 0.1124^2^ | 1.0000^2^ | 0.000 (0.000 – 1.451) |
| Total all journals | 11/60 (18%) | 13/58 (22%) | 0.6508^2^ | 1.0000^2^ | 1.284 (0.476 – 3.522) |
| ^1^*P-*values indicate the significance of the difference in proportion between samples before (before August 2003) and after (after June 2017) *JCN* and *Nature* introduced guidelines. *P*-values were determined with Fisher’s exact test (one-tailed, unless stated otherwise). Adjusted *P*-values were determined using the Holm-Bonferroni method.  ^2^ Two-tailed. | | | | | |

| ***Table S5.* Difference in percentage of articles that reports validation of all primary antibodies used between similar journals with and without guidelines. ^1^** | | | | | |
| --- | --- | --- | --- | --- | --- |
|  | ***n/N* (%)** | ***n/N* (%)** | ***P*-value** | **Adjusted *P-value*** | **Odds ratio (95% CI)** |
|  | ***JCN*** | ***Neuroscience*** |  |  |  |
| < Aug 2003 | 6/15 (40%) | 3/15 (20%) | 0.4270^2^ | 1.0000^2^ | 2.579 (0.411 – 20.449) |
| > June 2017 | 9/14 (64%) | 0/15 (0%) | 0.0002 | 0.0110 | INF (5.098 – INF) |
|  | ***Nature*** | ***Science*** |  |  |  |
| < Aug 2003 | 1/15 (7%) | 1/15 (7%) | 1.0000^2^ | 1.0000^2^ | 1.000 (0.012 – 83.978) |
| > June 2017 | 4/14 (29%) | 0/15 (0%) | 0.0421 | 1.0000 | INF (1.088 – INF) |
|  | **Total with guidelines** | **Total without guidelines** |  |  |  |
| < Aug 2003 | 7/30 (23%) | 4/30 (13%) | 0.5062^2^ | 1.0000^2^ | 1.956 (0.431 – 10.335) |
| > June 2017 | 13/28 (46%) | 0/30 (0%) | 1.186e-05 | 0.0007 | INF (6.499 – INF) |
| ^1^*P-*values indicate the significance of the difference in proportion between samples of similar journals (*JCN* and *Neuroscience*; *Nature* and Science) with (*JCN* and *Nature*) or without (*Neuroscience* and *Science*) guidelines. *P*-values were determined with Fisher’s exact test (one-tailed, unless stated otherwise). Adjusted P-values were determined using the Holm-Bonferroni method.  ^2^ Two-tailed. | | | | | |

# All primary antibodies basic information complete

| ***Table S6.* Difference in percentage of articles that reports complete basic information on all primary antibodies used** **before and after introduction of guidelines.^1^** | | | | | |
| --- | --- | --- | --- | --- | --- |
|  | **< Aug 2003**  *n/N* (%) | **> June 2017**  *n/N* (%) | ***P*-value** | **Adjusted *P-value*** | **Odds ratio (95% CI)** |
| *JCN* | 3/15 (20%) | 14/15 (93%) | 5.787e-05 | 0.0032 | 45.187 (5.507 - INF) |
| *Nature* | 0/15 (0%) | 5/13 (38%) | 0.0131 | 0.6286 | INF (1.756 – INF) |
| Total with guidelines | 3/30 (10%) | 19/28 (68%) | 5.235e-06 | 0.0003 | 17.799 (4.797 – INF) |
| *Neuroscience* | 2/15 (13%) | 5/15 (33%) | 0.3898^2^ | 1.0000^2^ | 3.125 (0.404 – 39.374) |
| *Science* | 1/15 (7%) | 3/14 (21%) | 0.3295^2^ | 1.0000^2^ | 3.652 (0.252 – 214.179) |
| Total without guidelines | 3/30 (10%) | 8/29 (28%) | 0.1042^2^ | 1.0000^2^ | 3.358 (0.696 – 22.071) |
| Total all journals | 6/60 (10%) | 27/57 (47%) | 9.247e-06^2^ | 0.0005^2^ | 7.947 (2.810 – 26.271) |
| ^1^*P-*values indicate the significance of the difference in proportion between samples before (before August 2003) and after (after June 2017) *JCN* and *Nature* introduced guidelines. *P*-values were determined with Fisher’s exact test (one-tailed, unless stated otherwise). Adjusted *P*-values were determined using the Holm-Bonferroni method.  ^2^ Two-tailed. | | | | | |

| ***Table S7.* Differences in percentage of articles that reports complete basic information on all primary antibodies used** **between similar journals with and without guidelines.^1^** | | | | | |
| --- | --- | --- | --- | --- | --- |
|  | ***n/N* (%)** | ***n/N* (%)** | ***P*-value** | **Adjusted *P-value*** | **Odds ratio (95% CI)** |
|  | ***JCN*** | ***Neuroscience*** |  |  |  |
| < Aug 2003 | 3/15 (20%) | 2/15 (13%) | 1.0000^2^ | 1.0000^2^ | 1.599 (0.154 – 22.300) |
| > June 2017 | 14/15 (93%) | 5/15 (33%) | 0.0009 | 0.0459 | 24.416 (3.170 – INF) |
|  | ***Nature*** | ***Science*** |  |  |  |
| < Aug2003 | 0/15 (0%) | 1/15 (7%) | 1.0000^2^ | 1.0000^2^ | 0.000 (0.000 – 39.001) |
| > June 2017 | 5/13 (38%) | 3/14 (21%) | 0.2928 | 1.0000 | 2.221 (0.413 – INF) |
|  | **Total with guidelines** | **Total without guidelines** |  |  |  |
| < Aug 2003 | 3/30 (10%) | 3/30 (10%) | 1.0000^2^ | 1.0000^2^ | 1.000 (0.123 – 8.150) |
| > June 2017 | 19/28 (68%) | 8/29 (28%) | 0.0025 | 0.1301 | 5.357 (1.848 – INF) |
| ^1^*P-*values indicate the significance of the difference in proportion between samples of similar journals (*JCN* and Neuroscience; *Nature* and Science) with (*JCN* and Nature) or without (*Neuroscience* and *Science*) guidelines. *P*-values were determined with Fisher’s exact test (one-tailed, unless stated otherwise). Adjusted P-values were determined using the Holm-Bonferroni method.  ^2^ Two-tailed. | | | | | |

# Change in type of validation

| ***Table S8.* Difference in percentage of validated articles reporting different types of validation before and after the introduction of guidelines.^1^** | | | | | | |
| --- | --- | --- | --- | --- | --- | --- |
|  | **Guidelines?** | **< August 2003**  **n/N (%)** | **> June 2017**  **n/N (%)** | ***P*-value** | **Adjusted *P*-value** | **Odds ratio (95% CI)** |
| Validation by reference to literature | With | 7/22 (32%) | 15/27 (56%) | 0.0845 | 1.0000 | 2.624 (0.859 – INF) |
|  | Without | 3/16 (19%) | 2/16 (13%) | 1.0000^2^ | 1.0000^2^ | - |
| Validation by reference to supplier | With | 0/23 (0%) | 7/26 (27%) | 0.0077 | 0.3752 | INF (1.962 – INF) |
|  | Without | 0/16 (0%) | 0/16 (0%) | - | - | - |
| Pre-adsorption | With | 4/20 (20%) | 4/23 (17%) | - | - | - |
|  | Without | 6/15 (40%) | 1/15 (7%) | 0.0959^2^ | 1.0000^2^ | - |
| Spatial localization | With | 3/20 (15%) | 7/23 (30%) | 0.2035 | 1.0000 | 2.428 (0.566 – INF) |
|  | Without | 3/15 (20%) | 1/15 (7%) | 0.6733^2^ | 1.0000^2^ | - |
| Secondary Ab without primary | With | 7/20 (35%) | 9/23 (39%) | 0.5154 | 1.0000 | 1.189 (0.355 – INF) |
|  | Without | 7/15 (47%) | 1/15 (7%) | 0.0475^2^ | 1.0000^2^ | - |
| Molecular weight in WB | With | 3/20 (15%) | 7/23 (30%) | 0.2035 | 1.0000 | 2.428 (0.566 – INF) |
|  | Without | 3/15 (20%) | 3/15 (20%) | - | - | - |
| ^1^*P-*values indicate the significance of the difference in proportion between samples before (before August 2003) and after (after June 2017) *JCN* and *Nature* introduced guidelines. *P*-values were determined with Fisher’s exact test (one-tailed, unless stated otherwise). Adjusted P-values were determined using the Holm-Bonferroni method.  ^2^ Two-tailed. | | | | | | |

| ***Table S9.* Differences in percentage of validated articles reporting different types of validation on all primary antibodies used** **between similar journals with and without guidelines. ^1^** | | | | | | |
| --- | --- | --- | --- | --- | --- | --- |
|  | **Before / after guidelines?** | **With guidelines *n/N* (%)** | **Without guidelines, *n/N* (%)** | ***P*-value** | **Adjusted *P-*value** | **Odds ratio (95% CI)** |
| Validation by reference to literature | < Aug 2003 | 7/22 (32%) | 3/16 (19%) | 0.4694^2^ | 1.0000^2^ | 1.986 (0.357 – 14.378) |
|  | > June 2017 | 15/27 (56%) | 2/16 (13%) | 0.0055 | 0.2734 | 8.317 (1.806 – INF) |
| Validation by reference to supplier | < Aug 2003 | 0/23 (0%) | 0/16 (0%) | - | - | - |
|  | > June 2017 | 7/26 (27%) | 0/16 (0%) | 0.0244 | 1.0000 | INF (1.338 – INF) |
| Pre-adsorption | < Aug 2003 | 4/20 (20%) | 6/15 (40%) | 0.2661^2^ | 1.0000^2^ | 0.386 (0.062 – 2.137) |
|  | > June 2017 | 4/23 (17%) | 1/15 (7%) | 0.3317 | 1.0000 | 2.874 (0.331 – INF) |
| Spatial localization | < Aug 2003 | 3/20 (15%) | 3/15 (20%) | 1.0000^2^ | 1.0000^2^ | 0.713 (0.081 – 6.272) |
|  | > June 2017 | 7/23 (30%) | 1/15 (7%) | 0.0852 | 1.0000 | 5.881 (0.803 – INF) |
| Secondary Ab without primary | < Aug 2003 | 7/20 (35%) | 7/15 (47%) | 0.5108^2^ | 1.0000^2^ | 0.624 (0.127 – 2.981) |
|  | > June 2017 | 9/23 (39%) | 1/15 (7%) | 0.0283 | 1.0000 | 8.560 (1.219 – INF) |
| Molecular weight in WB | < Aug 2003 | 3/20 (15%) | 3/15 (20%) | 1.0000^2^ | 1.0000^2^ | 0.713 (0.081 – 6.272) |
|  | > June 2017 | 7/23 (30%) | 3/15 (20%) | 0.3732 | 1.0000 | 1.725 (0.387 – INF) |
| ^1^*P-*values indicate the significance of the difference in proportion between samples of similar journals with (*JCN* and Nature) or without (*Neuroscience* and *Science*) guidelines. *P*-values were determined with Fisher’s exact test (one-tailed, unless stated otherwise). Adjusted P-values were determined using the Holm-Bonferroni method.  ^2^ Two-tailed. | | | | | | |
